# Cicatricial keratoconjunctivitis associated with lichen planus

**DOI:** 10.3205/oc000030

**Published:** 2015-10-07

**Authors:** Susan Huang, Prabjot Channa

**Affiliations:** 1 Department of Ophthalmology, Albert Einstein College of Medicine, Montefiore Medical Center, Bronx, NY, USA

**Keywords:** lichen planus, cicatricial keratoconjunctivitis, keratitis, cyclosporine

## Abstract

**Purpose:** To describe a case of cicatricial keratoconjunctivitis associated with lichen planus.

**Methods:** Case report.

**Results:** To our knowledge, this is the sixth reported case of cicatricial keratoconjunctivitis associated with lichen planus. A 73-year-old woman had persistent cicatricial keratoconjunctivitis. Histopathologic studies of the buccal mucosa biopsy specimen revealed lichen planus.

**Conclusion:** Lichen planus is a possible cause of cicatricial keratoconjunctivitis. Topical cyclosporine may stabilize the ocular surface, and additional systemic immunosuppression may be needed in severe cases. A correct diagnosis through biopsy is essential to start aggressive anti-inflammatory treatment to avoid vision loss.

## Introduction

Lichen planus (LP) is an autoimmune, inflammatory condition of unknown etiology that affects the skin and mucous membranes. Most commonly, the mouth and genitalia mucous membranes are involved [1]. Conjunctival involvement is rare, and may become indistinguishable from other forms of cicatricial conjunctivitis due to severe scarring that results. Even more rarely reported is corneal involvement. We report a case of lichen planus keratoconjunctivitis. 

## Case report

**Figure 1:**
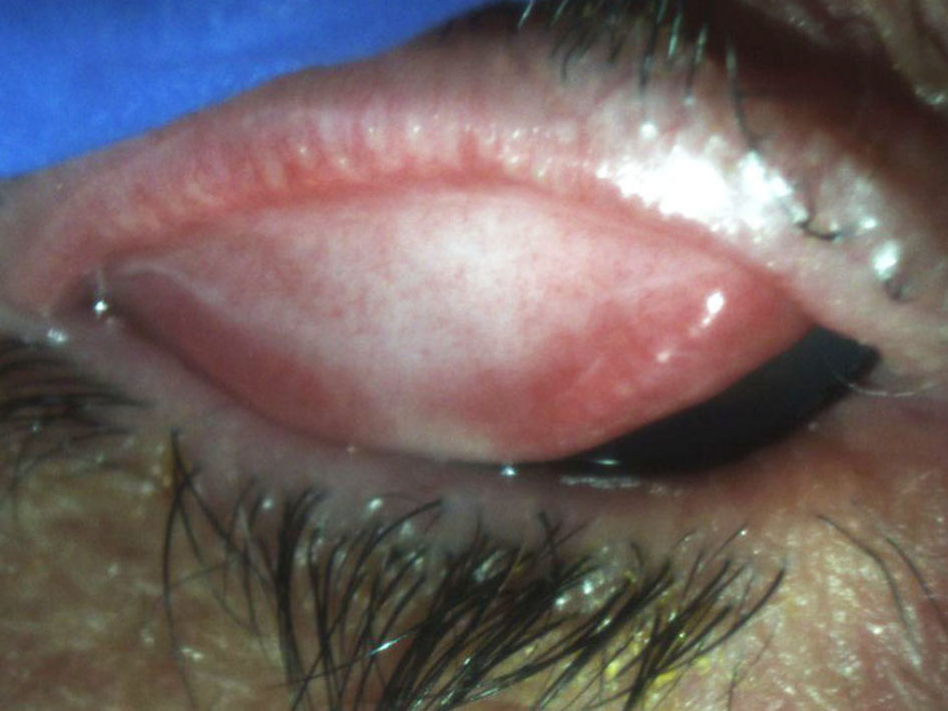
Subepithelial fibrosis of the right superior palpebral conjunctiva

**Figure 2:**
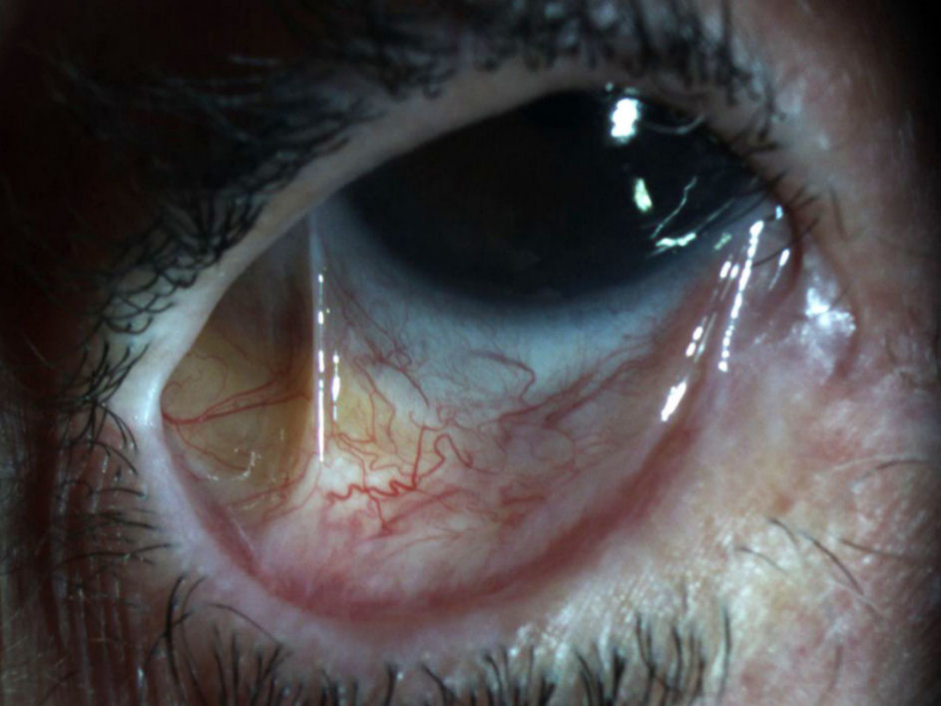
Foreshortening and symblepharon of the right lower palpebral conjunctiva

A 73-year-old Hispanic woman with a history of left eye enucleation secondary to endophthalmitis, history of dacryocystitis, and primary open-angle glaucoma presented with persistent right eye conjunctivitis and epithelial defect. On ophthalmic examination, her visual acuity was 20/50 in the right eye. The left eye had prosthesis. Intraocular pressure was within normal limits. The right eyelid showed thickened, telangectatic lid margins, with upper trichiasis and distichiasis. The semilunar fold was keratinized. There was subepithelial fibrosis of the right superior palpebral conjunctiva (Figure 1 (Fig. 1)) and symblepharon in the inferior medial palpebral conjunctiva (Figure 2 (Fig. 2)). The bulbar conjunctiva had notable amount of conjunctival injection. The cornea had superior pannus with diffuse severe punctuate epithelial erosions. 

Flat-topped polygonal papules were noted on the patient’s forearm. The patient was referred to Oral Surgery to perform buccal mucosal biopsy given the suspicion of the chronic conjunctivitis. A punch biopsy of the buccal mucosa was performed with histopathology confirming lichen planus, which was IgG-, IgA-, and C3-negative.

The patient was started on 0.05% cyclosporine eyedrop twice daily, very frequent lubrication with Artificial Tears, and Genteal Gel at bedtime. Later, 0.1% fluoromethalone acetate twice daily was added. Throughout the fourteen years that the patient was followed in our clinic, she had frequent epilation for her trichiasis. During this time course, she presented with several episodes of corneal abrasion and two episodes of corneal ulcer, which healed with topical antibiotic treatment. The cicatricial conjunctivitis remained grossly stable without further significant progression and with her best-corrected visual acuity minimally changed at 20/70 in her right eye. The option to start oral cyclosporine was discussed with the patient, but the patient deferred starting the medication due to the potential medication side effects and the very slow progression of her disease.

## Comment

Lichen planus is an autoimmune papulosquamous disease that affects the skin and mucous membranes. The etiology of LP is unclear, but is thought to be likely from T-cell mediated immunological response to an induced antigenic change in the basal membrane zone of mucosa or skin, triggering apoptosis of epithelial cells [1].

The cutaneous form of LP presents with a preference for the anterior aspect of the wrists and ankles and is characterized by violaceous, flat-topped polygonal papules with a superficial network of fine white lines [1]. Mucous membranes involvement may be associated with the cutaneous form and is characterized as reticular, erosive whitish macules, most typically on the buccal mucosa, lips, and genitalia. This form of LP is usually self-limiting, with spontaneous remission after one to two years. Whereas the second form of isolated mucosal LP may follow a chronic, unremitting course. Conjunctival involvement is rare, and usually presents as a chronic cicatricial conjunctivitis or keratoconjunctivitis [2]. Ocular involvement usually presents with other clinical manifestations of LP, but isolated conjunctival involvement has also been reported [3], [4], [5]. 

Histopathologic analysis of tissue samples, whether from conjunctiva or another body site, helps differentiate from other cicatricial conjunctivitis causes, such as mucous membrane pemphigoid, pemphigus vulgaris, Stevens-Johnson syndrome, graft-versus-host disease, linear IgA disease, and atopic keratoconjunctivitis, since they may be indistinguishable from clinical presentation alone [6], [7]. A definitive diagnosis is imperative because improper treatment would lead to progressive subepithelial fibrosis, trichiasis, entropion, corneal opacification, and keratoconjunctivitis sicca from chronic, persistent inflammation leading to severe vision loss [8]. 8.3% of pseudopemphigoid disorders with conjunctival involvement are actually caused by LP [6]. 

Lichen planus-associated keratitis has even more of a limited collection of published reports than conjunctival involvement, with only 5 previous reports to our knowledge based on a PubMed search of the English literature [5], [8], [9], [10], [11]. It is unclear whether corneal involvement is a direct effect of lichen planus, secondary to the abnormal tear film from conjunctival cicatricial changes, or a combination of both [9]. In our patient, trichiasis and ocular surface toxicity from chronic use of glaucoma eye drops may have also played a role. 

First-line treatment includes topical corticosteroid and cyclosporine. Aggressive use of preservative-free artificial tears is also important. If there is not a good response from topical treatments, systemic corticosteroids and immunosuppresants, such as cyclosporine, azathioprine, or mycophenolate mofetil can be used [3], [8], [12], [13], [14]. For severe disease state, amniotic membrane transplantation can be used [9].

Thus, our case reflects the need for accurate diagnosis through biopsy to distinguish this disease entity from other forms of cicatricial conjunctivitis or keratoconjunctivitis. A correct diagnosis is vital to start an aggressive anti-inflammatory treatment to avoid vision loss.

## Notes

### Patient consent

The patient gave informed consent for submission of this report to this journal. 

### Competing interests

The authors declare that they have no competing interests.
